# Economic stress in childhood and adulthood, and self-rated health: a population based study concerning risk accumulation, critical period and social mobility

**DOI:** 10.1186/1471-2458-12-761

**Published:** 2012-09-11

**Authors:** Martin Lindström, Kristina Hansen, Maria Rosvall

**Affiliations:** 1 Department of Clinical Sciences, Malmö University Hospital, Lund University, S-205 02, Malmö, Sweden; 2 Centre for Economic Demography, Lund University, Lund, Sweden

**Keywords:** Economic stress, self-rated health, Early life conditions, Life course perspective, Accumulation, Critical period, Social mobility, Social support, Trust, Sweden

## Abstract

**Background:**

Research in recent decades increasingly indicates the importance of conditions in early life for health in adulthood. Only few studies have investigated socioeconomic conditions in both childhood and adulthood in relation to health testing the risk accumulation, critical period, and social mobility hypotheses within the same setting. This study investigates the associations between economic stress in childhood and adulthood, and self-rated health with reference to the accumulation, critical period and social mobility hypotheses in life course epidemiology, taking demographic, social support, trust and lifestyle factors into account.

**Methods:**

The public health survey in Skåne (southern Sweden) in 2008 is a cross-sectional postal questionnaire study based on a random sample, in which 28,198 persons aged 18–80 years participated (55% participation). Logistic regression models were used to investigate associations between economic stress in childhood and adulthood, and self-rated health.

**Results:**

Three life-course socioeconomic models concerning the association between economic stress and self-rated health (SRH) were investigated. The results showed a graded association between the combined effect of childhood and adulthood economic stress and poor SRH in accordance with the accumulation hypothesis. Furthermore, upward social mobility showed a protecting effect and downward mobility increased odds ratios of poor SRH in accordance with the social mobility hypothesis. High/severe economic stress exposures in both stages of life were independently associated with poor SRH in adulthood. Furthermore, stratifying the study population into six age groups showed similar odds ratios of poor SRH regarding economic stress exposure in childhood and adulthood in all age groups among both men and women.

**Conclusions:**

The accumulation and social mobility hypotheses were confirmed. The critical period model was confirmed in the sense that both economic stress in childhood and adulthood had independent effects on poor SRH. However, it was not confirmed in the sense that a particular window in time (in childhood or adulthood) had a specifically high impact on self-rated health.

## Background

In recent decades there has been a dramatic increase in life course epidemiology research [1,2]. Research investigating the specific notion within life course epidemiology that socioeconomic differences in disease risk in adulthood may be caused by socioeconomic differences in risk exposure in childhood and adolescence [3] has surged accordingly. Many of the studies in this field of research have concerned cardiovascular diseases and issues related to cardiovascular diseases [4-8], but the effects of socioeconomic circumstances in early life on health and risk of poor health have also been investigated in studies with adult self-rated health as the outcome [9-11].

The idea that risk exposure early in life will have health consequences later in life was first supported empirically in 1934 by Kermack et al. who demonstrated that lower mortality in England, Scotland and Sweden were primarily associated with year of birth (birth cohort), and to a lesser extent with conditions at the time point when death occurred [12]. Beginning with mostly ecological studies in the 1970s and 1980s, the notion that biological, behavioural, social and other conditions in early life may influence health as well as morbidity and mortality later in life has been increasingly empirically researched. This research first concerned effects of conditions during life in utero on the development of the metabolic syndrome and cardiovascular diseases during adulthood. Empirical results from these studies generated the hypothesis that the last trimester constitutes a critical period of “programming” cells and organs to yield e.g. hypertension, obesity, high blood cholesterol, deranged composition of cholesterol fractions and higher blood triglycerides which lead to the metabolic syndrome [13]. However, the critical period concept more broadly refers to any stage in the individual’s development in which a heightened sensitivity to risk factors or protective factors may have effects on health in later life [14].

The critical period hypothesis is still being investigated [15,16], but two other life course hypotheses have also been forwarded and investigated in recent decades. The accumulation of risk hypothesis proposes that exposures accumulate over the life course and cumulatively increase the risk of chronic disease and mortality in a graded manner [14,17,18]. The social mobility hypothesis is more specific to life course *social* epidemiology. It suggests that intra- and inter-generational social mobility, mostly defined in terms of socioeconomic status (SES) defined according to occupational status, will affect health later in life and should be regarded as potentially important as a social cause of disease [19].

Despite the existence of at least three socioeconomic models concerning the causal connection between socioeconomic conditions in early life, socioeconomic conditions in adulthood, and disease risk in adulthood only few empirical studies have investigated all three models on the same population in the same study. These studies concern cardiovascular mortality [14,20] and total mortality [20]. To our knowledge, no study testing the three life course hypotheses concerning socioeconomic differences in health measured as self-rated health has been published.

In this study the associations between economic stress during childhood, economic stress in adulthood and self-rated health will be investigated. Economic stress in childhood has been less investigated than socioeconomic position of the father or both parents during childhood [10]. However, economic stress within the family is associated with mortality and morbidity [21], infant mortality [22], poor mental health [23], and lack of sense of well-being [24]. self-rated health is a good predictor of future morbidity and mortality, e.g. for incidence of cardiovascular diseases [25,26]. Emotional support, instrumental support and generalized trust in other people, the latter often regarded as an aspect of social capital [27], are associated with self-rated health and thus adjusted for as confounders [28]. Daily smoking and high alcohol consumption may be regarded as mediating factors in the chain of causality between economic stress and health.

The aim of this study is to investigate the associations between economic stress in both childhood and adulthood, and self-rated health with reference to the accumulation, critical period and social mobility hypotheses in life course epidemiology, taking demographic, psychosocial and lifestyle factors into account.

## Methods

### Study population

The 2008 public health survey in Skåne in southern Sweden is a cross sectional study. A total of 28,198 men and women randomly selected from the official population registers of people living in Skåne born between 1928 and 1990 answered a postal questionnaire in the period August and September 2008, which represents a 55% response rate. Two letters of reminder were sent. This study has been approved by the Ethical Committee at Lund University, Sweden.

### Definitions

#### Dependent variable

*Self-rated health* was investigated with the question “How do you rate your general health status?” with the five optional answers “very good”, “good”, “neither good nor poor”, “poor” and “very poor”. The answers were dichotomised into good (the two first alternatives) and poor (the three latter alternatives) health.

### Independent variables

*Age* was categorised into the age groups 18–24, 25–34, 35–44, 45–54, 55–64 and 65–80 years.

All analyses included *sex*, but no stratification for sex was conducted in the multiple analyses because the distributions of both the outcome and exposure variables and their associations were very similar according to sex.

*Born in Sweden/born in other country than Sweden.* All participants born in other countries than Sweden were collapsed into a single category that was compared with the category born in Sweden.

*Socioeconomic status (SES)* by occupation includes the six categories on the labour market: higher non-manual employees, medium level non-manual employees, low level non-manual employees, skilled manual workers and unskilled manual workers as well as self-employed/farmers. The groups outside the workforce comprise old age pensioners above age 65 years, early retired (retired before age 65 for reasons of health or early retirement entitlement in the employment contract), unemployed, students, persons on long term sick leave and unclassified.

*Emotional support* was assessed with the item “Do you feel that you have somebody or some persons who can given you proper personal support to cope with the stress and problems of life?” It has four options: “Yes, I am absolutely certain to get such support”, “Yes, possibly”, “”Not certain”, and “No”. The three latter alternatives were defined as low emotional support.

*Instrumental support* was assessed with the item “Can you get help from somebody or some persons in case of disease or practical problems (borrowing what minor things that you need, help with reparation, help to write a an official letter, advice or information)?” It has the same optional answers as the emotional support item and was dichotomized correspondingly.

*Generalized (horizontal) trust in other people* assesses the individual’s perception of generalized trust in other people with the item “Generally, you can trust other people” with the four alternative answers: “Do not agree at all”, “Do not agree”, “Agree”, and “Completely agree”. These alternatives were dichotomized with the two first indicating low trust and the two latter high.

The generalized trust in other people item has been used in the same manner internationally [27,29].

*Daily smoking* was assessed with the item “Do you smoke?” with the options “daily smoker”, “smoker, but not daily”, “never smoked” and “non-smoker, stopped smoking”. The variable was dichotomized by collapsing the three latter options.

*Risk and high risk alcohol consumption* was defined according to international recommendations [30] as 128.0g 100% alcohol per week for men and 96.0g per week for women. The amount of alcohol was assessed with a QF (quantity/frequency) method which combines the number of days of alcohol consumption and the amount of alcohol consumed during such a typical day (beer, wine, liquor) during a 30 days period (the past 30 days), and the number of days in the past year with a day consumption of 37cl strong liquor or more, four cans of beer or more or one bottle of wine (75cl) or more (with half the amount for women). Alcohol consumption is thus a dichotomous variable, and abstainers are included in the below risk level consumption category in this study.

*Economic stress in childhood* was assessed with the item “Did your family experience economic hardship when you grew up?” with the three alternatives “No, no significant problems” (1), “Yes, less severe problems and/or problems during short time periods” (2) and “Yes, severe problems and/or problems during long time periods” (3).

*Economic stress in adulthood* (current situation) was assessed with the item “How often during the past twelve months have you had problems paying your bills (rent, electricity, interest, mortgages, insurances etc.)?” with the four alternative answers “never” (1), “occasionally” (2), “every second month” and “every month”. The two latter options “every second month” and “every month” were collapsed (3), which yielded three alternatives in the analyses in this study.

**Figure 1:**
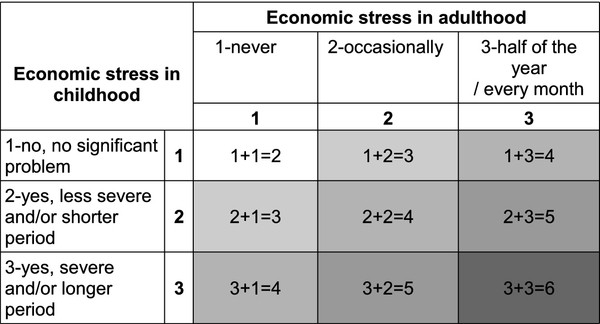
**The accumulation hypothesis. **Accumulation of risk exposure in a graded model. Accumulation results may range from the most favourable 2(1 + 1) to the least favourable 6 (3 + 3), which yields five accumulation categories.

**Figure 2:**
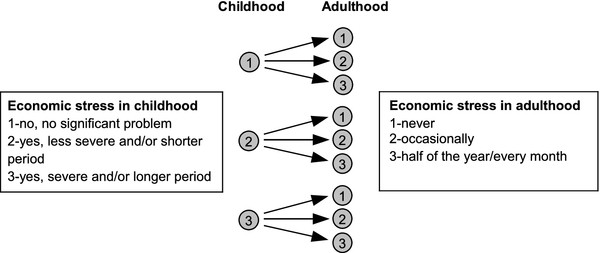
**The social mobility hypothesis. **Baseline economic stress in childhood and the economic stress mobility opportunities in adulthood.

*Economic stress in childhood* and *economic stress in adulthood* (current situation) were analyzed combined to address the three hypotheses concerning accumulation, critical period and social mobility. The accumulation hypothesis was investigated by adding the exposure to economic stress in childhood and adulthood: respondents with no economic stress in childhood (1) as well as no economic stress in adulthood (1) being the most optimal combination (1 + 1), respondents with no problems in either childhood or adulthood combined with lesser (medium) problems in either childhood or adulthood being the second best combination (1 + 2 or 2 + 1). The (1 + 3), (3 + 1), (2 + 2) combinations were analyzed collapsed into a third category, and the (2 + 3) and (3 + 2) combinations were analyzed collapsed into a fourth category. The least optimal combination was severe economic stress in both childhood and adulthood (3 + 3). This yielded a total of five possible accumulation of risk combinations. The accumulation hypothesis is illustrated in Figure 1. The critical period hypothesis was tested by including both economic stress in childhood and economic stress in adulthood as two separate and categorized variables in the same model. The social mobility hypothesis was investigated by analyzing the mobility from no economic problems in childhood to either no problems, less frequent problems or severe problems in adulthood. The baseline economic stress in childhood among respondents with less severe problems and/or problems during short time periods as well as with severe problems and/or problems during long time periods were analyzed similarly with the economic stress in childhood information as baseline. Inter-generational social mobility was defined as a different economic stress situation in adult life than in childhood indicated by presence of economic stress of the parents at that time. Upwardly and downwardly mobile subjects were compared with those who had a similar chance of mobility from the same initial social position but did not move. The social mobility hypothesis is illustrated in Figure 2.

### Statistics

**Figure 3:**
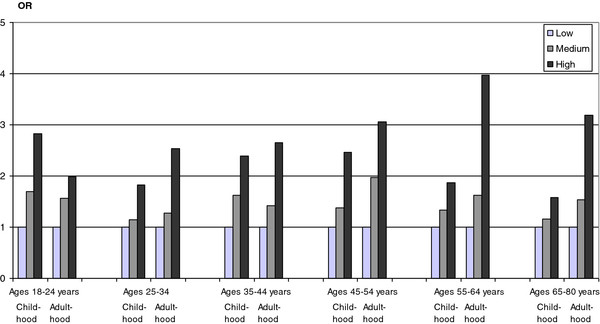
**Odds ratios (ORs) of poor self-rated health in relation to exposure to economic stress in childhood and adulthood, respectively, stratified by age in *men*, the Scania Public Health survey 2008. **Economic stress was categorized into: low, medium and high levels of economic stress. Low (childhood), i.e., no significant problems with economic hardship in the family during grow-up; Low (adulthood), i.e., never problems with paying bills during the past 12 months; Medium (childhood), i.e., less severe problems and/or problems during short time periods with economic hardship in the family during grow-up; Medium (adulthood), i.e., occasionally problems with paying bills during the past 12 months; High (childhood), i.e., severe problems with economic hardship in the family during grow-up; High (adulthood), i.e., at least every second month problems with paying bills during the past 12 months.

**Figure 4:**
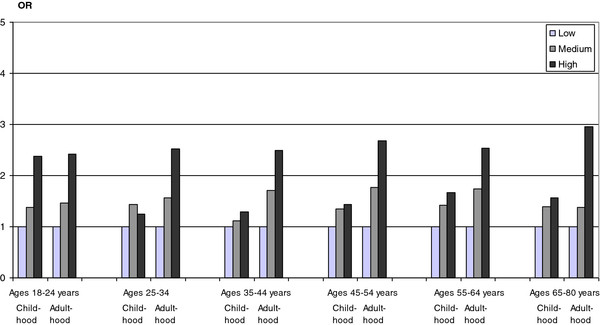
**Odds ratios (ORs) of poor self-rated health in relation to exposure to economic stress in childhood and adulthood, respectively, stratified by age in *women*, the Scania Public Health survey 2008. **Economic stress was categorized into: low, medium and high levels of economic stress. Low (childhood), i.e., no significant problems with economic hardship in the family during grow-up; Low (adulthood), i.e., never problems with paying bills during the past 12 months; Medium (childhood), i.e., less severe problems and/or problems during short time periods with economic hardship in the family during grow-up; Medium (adulthood), i.e., occasionally problems with paying bills during the past 12 months; High (childhood), i.e., severe problems with economic hardship in the family during grow-up; High (adulthood), i.e., at least every second month problems with paying bills during the past 12 months.

Prevalences (%) of poor self-rated health, age, country of birth, socioeconomic status, emotional support, instrumental support, trust, daily smoking, high alcohol consumption, economic stress in childhood and economic stress in adulthood stratified by sex were calculated (Table 1). Prevalences (%) and odds ratios with 95% confidence intervals (OR:s, 95% CI) of poor self-rated health were calculated according to age, country of birth, socioeconomic status, emotional support, instrumental support, trust, daily smoking, high alcohol consumption, economic stress in childhood and economic stress in adulthood, stratified by sex, were calculated (Table 2). Crude, age-adjusted and multiple adjusted odds ratios and 95% confidence intervals of poor self-rated health according to the accumulation hypothesis were calculated (Table 3). Crude, age-adjusted and multiple adjusted odds ratios and 95% confidence intervals of poor self-rated health according to the critical period hypothesis were calculated (Table 4). Multiple adjusted odds ratios (and 95% confidence intervals) of poor self-rated health according to the critical period hypothesis were also calculated in models stratified for age (each of the six age intervals) among men (Figure 3) and women (Figure 4). Crude, age-adjusted and multiple adjusted odds ratios and 95% confidence intervals of poor self-rated health according to the social mobility hypothesis were calculated (Table 5). All statistical analyses in Tables 2, 3, 4, and 5 and Figures 3, 4 were conducted in logistic regression models. The statistical analyses were performed using the PASW software package version 18.0 [31].

**Table 1:** Prevalence (%) of self-rated health, demographic characteristics, socioeconomic status (SES), emotional support, instrumental support, trust in other people, daily smoking, alcohol consumption, and economic stress in childhood and adulthood. Men (n = 12,726), women (n = 15,472), and total (n = 28,198). The public health survey in Skåne 2008

	**Men (n = 12,726)**	**Women (n = 15,472)**	**Total (n = 28,198)**
**Self rated health**			
Good	72.6	70.0	71.2
Poor	27.4	30.0	28.8
(Missing)	(250)	(396)	(646)
**Age**			
18-24	8.3	9.1	8.8
25-34	12.3	13.9	13.2
35-44	16.4	17.2	16.9
45-54	17.7	18.5	18.1
55-64	21.2	19.3	20.1
65-80	24.2	21.9	22.9
(Missing)	(0)	(0)	(0)
**Country of birth**			
Sweden	86.1	85.9	86.0
Other country	13.9	14.1	14.0
(Missing)	(273)	(282)	(555)
**Socioeconomic status**			
Higher non-manual	10.2	8.1	9.1
Medium non-manual	12.0	16.3	14.3
Lower non-manual	4.8	9.5	7.4
Skilled manual	10.7	8.7	9.6
Unskilled manual	11.6	11.1	11.3
Self-employed/farmer	7.7	3.7	5.5
Early retired	3.2	4.6	4.0
Unemployed	3.2	3.4	3.3
Student	4.9	6.7	5.9
Old age pensioner	26.2	23.2	24.6
Unclassified	4.7	3.4	4.0
Long term sick leave	0.9	1.3	1.1
(Missing)	(212)	(244)	(456)
**Emotional support**			
High	62.8	69.6	66.6
Low	37.2	30.4	33.4
(Missing)	(289)	(357)	(646)
**Instrumental support**			
High	71.3	76.6	74.2
Low	28.7	23.4	25.8
(Missing)	(295)	(338)	(633)
**Trust (horizontal)**			
High	66.1	64.3	65.2
Low	33.9	35.7	34.8
(Missing)	(522)	(685)	(1207)
**Daily smoking**			
No	87.4	85.1	86.1
Yes	12.6	14.9	13.9
(Missing)	(169)	(184)	(353)
**Alcohol consumption**			
Non-risk	78.4	89.9	84.5
Risk-high risk	21.6	10.1	15.5
(Missing)	(1397)	(2513)	(3910)
**Economic stress in childhood**			
No significant problem	63.2	62.5	62.8
Less severe and/or shorter period	27.1	27.7	27.4
Severe and/or longer period	9.7	9.7	9.7
(Missing)	(341)	(354)	(695)
**Economic stress in adulthood**			
Never	79.5	76.5	77.8
Occasionally	14.1	15.7	15.0
Half the year	3.1	3.6	3.4
Every month	3.3	4.2	3.8
(Missing)	(307)	(335)	(642)

**Table 2:** Prevalence (%) and odds ratios (OR, 95% CI) in bivariate analyses of poor self-rated health according to age, country of birth, socioeconomic status (SES), emotional support, instrumental support, trust in other people (horizontal trust), daily smoking, alcohol consumption, and economic stress in childhood and adulthood. Men (n = 12,726) and women (n = 15,472). The public health survey in Skåne 2008

	**Men (n = 12,726)**		**Women (n = 15,472)**	
	**%**	**OR(95%CI)**	**%**	**OR(95%CI)**
**Age**				
18-24	13.7	1.00	21.2	1.00
25-34	15.2	1.12 (0.93-1.34)	20.0	0.93 (0.80-1.08)
35-44	20.4	1.60 (1.35-1.90)	24.3	1.19 (1.03-1.38)
45-54	31.6	2.89 (2.45-3.41)	30.2	1.60 (1.39-1.86)
55-64	35.8	3.49 (2.97-4.11)	38.6	2.33 (2.02-2.69)
65-80	40.5	4.27 (3.63-5.02)	44.5	2.98 (2.59-3.42)
(Missing)	(250)		(396)	
**Country of birth**				
Sweden	25.7	1.00	28.1	1.00
Other country	32.1	1.37 (1.25-1.50)	36.8	1.49 (1.36-1.63)
(Missing)	(456)		(616)	
**Socioeconomic status**				
Higher non-manual	14.2	1.00	14.5	1.00
Medium non-manual	13.2	0.91 (0.74-1.13)	16.8	1.20 (0.97-1.48)
Lower non-manual	22.3	1.72 (1.36-2.18)	22.3	1.70 (1.37-2.11)
Skilled manual	24.4	1.94 (1.60-2.36)	24.5	1.92 (1.54-2.38)
Unskilled manual	25.0	2.00 (1.65-2.42)	28.0	2.30 (1.87-2.82)
Self-employed/farmer	19.7	1.48 (1.19-1.83)	17.8	1.28 (0.97-1.70)
Early retired	81.4	26.11 (19.79-34.44)	83.8	30.44 (23.08-40.14)
Unemployed	42.3	4.40 (3.50-5.55)	41.8	4.24 (3.33-5.40)
Student	15.6	1.11 (0.88-1.40)	19.7	1.45 (1.16-1.82)
Old age pensioner	39.1	3.87 (3.25-4.61)	44.0	4.65 (3.84-5.63)
Unclassified	19.5	1.45 (1.16-1.83)	23.1	1.77 (1.37-2.30)
Long term sick leave	89.9	52.90 (28.67-97.60)	89.7	51.27 (30.92-85.04)
(Missing)	(417)		(605)	
**Emotional support**				
High	20.8	1.00	23.4	1.00
Low	37.1	2.25 (2.08-2.43)	43.5	2.52 (2.33-2.72)
(Missing)	(466)		(686)	
**Instrumental support**				
High	22.0	1.00	24.5	1.00
Low	39.1	2.28 (2.10-2.47)	45.3	2.55 (2.35-2.76)
(Missing)	(473)		(665)	
**Trust (horizontal)**				
High	22.0	1.00	23.5	1.00
Low	34.0	1.82 (1.69-1.97)	38.4	2.02 (1.88-2.18)
(Missing)	(706)		(1010)	
**Daily smoking**				
No	24.4	1.00	27.9	1.00
Yes	42.1	2.25 (2.03-2.48)	40.3	1.75 (1.59-1.93)
(Missing)	(351)		(518)	
**Alcohol consumption**				
Non-risk	24.9	1.00	27.0	1.00
Risk-high risk	27.3	1.13 (1.03-1.25)	27.5	1.02 (0.89-1.16)
(Missing)	(1541)		(2783)	
**Economic stress in childhood**				
No significant problem (1)	21.9	1.00	24.9	1.00
Less severe and/or shorter period (2)	31.8	1.66 (1.52-1.81)	35.7	1.67 (1.54-1.81)
Severe and/or longer period (3)	44.5	2.85 (2.53-3.22)	43.0	2.27 (2.02-2.56)
(Missing)	(490)		(690)	
**Economic stress in adulthood**				
Never (1)	23.5	1.00	25.9	1.00
Occasionally (2)	32.4	1.56 (1.40-1.72)	34.1	1.48 (1.34-1.62)
Half the year (3)	45.5	2.71 (2.25-3.27)	44.4	2.28 (1.92-2.72)
Every month (3)	54.6	3.89 (3.26-4.65)	58.6	4.04 (3.43-4.76)
(Missing)	(491)		(669)	

**Table 3:** Prevalence (%) and odds ratios (OR, 95% CI) in crude, age-adjusted and multiple adjusted analyses of poor self rated health according to economic stress risk accumulation (childhood + adulthood combined). Men (n = 12,726) and women (n = 15,472). The public health survey in Skåne 2008

**Risk accumulation**	**%**	**OR(95% CI)**^**a**^	**OR(95% CI)**^**b**^	**OR(95% CI)**^**c**^
Lowest (1 + 1)	21.0	1.00	1.00	1.00
(1 + 2) or (2 + 1)	29.1	1.55 (1.45-1.65)	1.54 (1.45-1.65)	1.59 (1.49-1.70)
(1 + 3), (2 + 2) or (3 + 1)	40.1	2.52 (2.34-2.72)	2.51 (2.33-2.71)	2.78 (2.57-3.00)
(2 + 3) or (3 + 2)	48.7	3.60 (3.20-4.05)	3.58 (3.18-4.03)	4.41 (3.90-4.99)
Highest (3 + 3)	61.5	5.99 (4.91-7.32)	5.98 (4.90-7.31)	7.40 (6.02-9.10)
	**OR(95% CI)**^**d**^	**OR(95% CI)**^**e**^	**OR(95% CI)**^**f**^	**OR(95% CI)**^**g**^
Lowest (1 + 1)	1.00	1.00	1.00	1.00
(1 + 2) or (2 + 1)	1.57 (1.48-1.68)	1.56 (1.45-1.66)	1.49 (1.39-1.59)	1.46 (1.36-1.57)
(1 + 3), (2 + 2) or (3 + 1)	2.69 (2.49-2.91)	2.54 (2.35-2.75)	2.37 (2.18-2.56)	2.32 (2.14-2.52)
(2 + 3) or (3 + 2)	4.26 (3.77-4.82)	3.98 (3.51-4.51)	3.48 (3.06-3.95)	3.37 (2.96-3.83)
Highest (3 + 3)	6.95 (5.64-8.56)	6.29 (5.10-7.76)	5.48 (4.42-6.80)	5.37 (4.32-6.68)
	**OR(95% CI)**^**h**^	**OR(95% CI)**^**i**^	**OR(95% CI)**^**j**^	
Lowest (1 + 1)	1.00	1.00	1.00	
(1 + 2) or (2 + 1)	1.43 (1.33-1.53)	1.40 (1.30-1.50)	1.36 (1.26-1.47)	
(1 + 3), (2 + 2) or (3 + 1)	2.20 (2.02-2.39)	2.11 (1.94-2.30)	2.01 (1.84-2.20)	
(2 + 3) or (3 + 2)	3.18 (2.78-3.63)	2.98 (2.60-3.40)	2.83 (2.44-3.28)	
Highest (3 + 3)	4.81 (3.85-6.02)	4.40 (3.52-5.51)	5.24 (4.01-6.83)	

a Crude.

b Adjusted for sex.

c Adjusted for sex and age.

e Adjusted for sex, age, country of birth and socioeconomic status.

f Adjusted for sex, age, country of birth, socioeconomic status and emotional support.

g Adjusted for sex, age, country of birth, socioeconomic status, emotional support and instrumental support.

h Adjusted for sex, age, country of birth, socioeconomic status, emotional support, instrumental support and trust.

i Adjusted for sex, age, country of birth, socioeconomic status, emotional support, instrumental support, trust and daily smoking.

j Adjusted for sex, age, country of birth, socioeconomic status, emotional support, instrumental support, trust, daily smoking and risk consumption of alcohol.

**Table 4:** Odds ratios (OR, 95% CI) in crude, age-adjusted and multiple adjusted analyses of poor self rated health according to economic stress critical period (childhood + adulthood included as separate variables in the same model). Men (n = 12,726) and women (n = 15,472). The public health survey in Skåne 2008

**Critical period**	**OR(95% CI)**^**a**^	**OR(95% CI)**^**b**^	**OR(95% CI)**^**c**^	**OR(95% CI)**^**d**^
**Economic stress in childhood**				
No significant problem (1)	1.00	1.00	1.00	1.00
Less severe and/or shorter period (2)	1.55 (1.46-1.65)	1.55 (1.46-1.65)	1.48 (1.39-1.57)	1.46 (1.37-1.55)
Severe and/or longer period (3)	2.27 (2.08-2.48)	2.27 (2.09-2.48)	2.05 (1.87-2.24)	1.96 (1.78-2.14)
**Economic stress in adulthood**				
Never (1)	1.00	1.00	1.00	1.00
Occasionally (2)	1.42 (1.32-1.52)	1.41 (1.31-1.51)	1.89 (1.75-2.04)	1.87 (1.73-2.02)
Half the year/every month (3)	2.84 (2.59-3.12)	2.83 (2.58-3.10)	3.80 (3.45-4.18)	3.73 (3.38-4.11)
	**OR(95% CI)**^**e**^	**OR(95% CI)**^**f**^	**OR(95% CI)**^**g**^	**OR(95% CI)**^**h**^
**Economic stress in childhood**				
No significant problem (1)	1.00	1.00	1.00	1.00
Less severe and/or shorter period (2)	1.44 (1.35-1.53)	1.37 (1.28-1.46)	1.36 (1.27-1.45)	1.34 (1.25-1.43)
Severe and/or longer period (3)	1.88 (1.72-2.07)	1.79 (1.63-1.97)	1.80 (1.63-1.97)	1.73 (1.57-1.91)
**Economic stress in adulthood**				
Never (1)	1.00	1.00	1.00	1.00
Occasionally (2)	1.83 (1.69-1.97)	1.73 (1.60-1.87)	1.71 (1.58-1.85)	1.66 (1.53-1.80)
Half the year/every month (3)	3.44 (3.12-3.79)	3.16 (2.86-3.49)	3.06 (2.76-3.38)	2.86 (2.58-3.17)
	**OR(95% CI)**^**i**^	**OR(95% CI)**^**j**^		
**Economic stress in childhood**				
No significant problem (1)	1.00	1.00		
Less severe and/or shorter period (2)	1.32 (1.24-1.42)	1.29 (1.20-1.39)		
Severe and/or longer period (3)	1.70 (1.54-1.87)	1.65 (1.48-1.84)		
**Economic stress in adulthood**				
Never (1)	1.00	1.00		
Occasionally (2)	1.59 (1.47-1.73)	1.55 (1.42-1.69)		
Half the year/every month (3)	2.68 (2.41-2.98)	2.78 (2.48-3.13)		

a Crude.

b Adjusted for sex.

c Adjusted for sex and age.

d Adjusted for sex, age and country of birth.

e Adjusted for sex, age, country of birth and socioeconomic status.

f Adjusted for sex, age, country of birth, socioeconomic status and emotional support.

g Adjusted for sex, age, country of birth, socioeconomic status, emotional support and instrumental support.

h Adjusted for sex, age, country of birth, socioeconomic status, emotional support, instrumental support and trust.

i Adjusted for sex, age, country of birth, socioeconomic status, emotional support, instrumental support, trust and daily smoking.

j Adjusted for sex, age, country of birth, socioeconomic status, emotional support, instrumental support, trust, daily smoking and risk consumption of alcohol.

**Table 5:** Prevalence (%) and odds ratios (OR, 95% CI) in crude, age-adjusted and multiple adjusted analyses of poor self rated health according to social mobility (childhood to adulthood). The public health survey in Skåne 2008

**Social mobility (childhood-adulthood)**	**%**	**OR(95% CI)**^**a**^	**OR(95% CI)**^**b**^	**OR(95% CI)**^**c**^
**No-Never (1 to 1)**	21.0	1.00	1.00	1.00
**No-occasionally (1 to 2)**	27.4	1.42 (1.28-1.57)	1.86 (1.67-2.07)	1.51 (1.34-1.70)
**No-half the year/every month (1 to 3)**	47.3	3.39 (2.96-3.87)	4.32 (3.76-4.96)	2.90 (2.46-3.42)
**(N = 16878)**				
**Social mobility (childhood-adulthood)**	**%**	**OR(95% CI)**^**a**^	**OR(95% CI)**^**b**^	**OR(95% CI)**^**c**^
**Less severe-Never (2 to 1)**	29.9	0.71 (0.63-0.80)	0.51 (0.45-0.58)	0.62 (0.54-0.72)
**Less severe-occasionally (2 to 2)**	37.4	1.00	1.00	1.00
**Less severe-half the year/every month (2 to 3)**	50.3	1.70 (1.42-2.02)	1.69 (1.41-2.03)	1.52 (1.22-1.89)
**(N = 7386)**				
**Social mobility (childhood-adulthood)**	**%**	**OR(95% CI)**^**a**^	**OR(95% CI)**^**b**^	**OR(95% CI)**^**c**^
**Severe-Never (3 to 1)**	38.4	0.39 (0.32-0.49)	0.29 (0.23-0.36)	0.31 (0.23-0.42)
**Severe-occasionally (3 to 2)**	46.6	0.55 (0.42-0.71)	0.51 (0.39-0.67)	0.48 (0.34-0.67)
**Severe-half the year/every month (3 to 3)**	61.4	1.00	1.00	1.00
**(N = 2592)**				

a Crude.

b Adjusted for sex and age.

c Adjusted for sex, age, country of birth, socioeconomic status, emotional support, instrumental support, trust, daily smoking and risk consumption of alcohol

26856 respondents included in analyses, 1342 respondents missing values.

## Results

Table 1 shows that 27.4% of the men and 30.0% of the women had poor self-rated health. The prevalence of age, country of birth, socioeconomic status, emotional support, instrumental support, trust, daily smoking, high alcohol consumption, economic stress in childhood and economic stress in adulthood variables among men and women are also displayed in Table 1.

Table 2 shows that poor self-rated health was more common among the middle-aged and old, those born abroad, those with low socioeconomic status, unemployed, sick leave pensioners, low emotional support, low instrumental support, low trust, daily smoking, high alcohol consumption, economic stress in childhood and economic stress in adulthood.

Table 3 shows that the crude odds ratios of poor self-rated health were 1.55 (1.45-1.65) in the (1 + 2, 2 + 1) accumulation group, 2.52 (2.34-2.72) in the (1 + 3, 3 + 1, 2 + 2) accumulation group, 3.60 (3.20-4.05) in the (2 + 3, 3 + 2) accumulation group and 5.99 (4.91-7.32) in the (3 + 3) accumulation group compared to the reference (1 + 1) no lifecourse economic stress accumulation group. These odds ratios remained approximately unaltered throughout the multiple analyses (see Table 3).

Table 4 shows that the crude odds ratios of poor self-rated health according to economic stress in childhood and adulthood were significant compared to the no stress alternatives, respectively, when included in the same logistic regression model. While the odds ratios of poor self-rated health for economic stress in childhood (categories 2 and 3) were significant in the crude model compared to the no economic stress in childhood reference group (category 1), the odds ratios of poor self-rated health according to economic stress in adulthood (categories 2 and 3) were also significant and even higher compared to the no economic stress in adulthood group (category 1). The odds ratios of poor self-rated health according to economic stress in childhood (categories 2 and 3) decreased successively compared to the no stress in childhood reference group (category 1) as more variables were included in the multiple logistic regression model, while, on the other hand, the odds ratios according to economic stress in adulthood (categories 2 and 3) did not decrease compared to the no stress in adulthood reference group (category 1). Figures 3 and 4 displays the odds ratios of poor self-rated health according to exposure to economic stress in childhood and adulthood, respectively, stratified by age in men (Figure 3) and women (Figure 4). All association between economic stress in childhood and adulthood, respectively, and poor self-rated health were statistically significant at the 5% significance level, the only exception being the association between economic stress in childhood and poor self-rated health among women aged 35–44 years (95% confidence intervals not shown in Figures 3– 4).

Inter-generational social mobility was defined as a different economic stress situation in adult life than in childhood (indicated by presence of economic stress of the parents at that time). Upwardly and downwardly mobile groups of respondents were compared with those who had a similar chance of mobility from the same initial social position but did not move. Table 5 shows the association between inter-generational social mobility and self-rated health. Compared with subjects who were without economic stress at both stages in life, subjects with downward intergenerational social mobility showed higher odds of poor self-rated health, i.e., OR =1.42 (95% CI: 1.28, 1.57) for those occasionally having problems paying their bills, and OR =3.39 (95% CI: 2.96, 3.87) for those with more frequent problems. Similarly, socially upwardly mobile subjects showed lower odds of poor self-rated health than subjects who were exposed to the highest levels of economic stress during both childhood and adulthood. Compared with those having less severe economic problems during childhood and with occasional problems in adulthood, socially upwardly mobile subjects showed lower odds of poor self-rated health, while those who were socially downwardly mobile showed higher odds of poor self-rated health.

## Discussion

Three life-course socioeconomic models concerning the association between economic stress and self-rated health were investigated in the same population. The accumulation hypothesis was confirmed because the results showed a graded association between the combined effect of childhood and adulthood and poor self-rated health. The social mobility hypothesis was confirmed because there was a protecting or negative effect on poor SRH depending on the mobility direction, whether it was up- or downward. Upward social mobility showed a protecting effect and downward mobility increased odds ratios of poor SRH. The critical period model was confirmed in the sense that both economic stress in childhood and economic stress in adulthood seemed to be of importance to self-rated health measured in adulthood. However, it was not confirmed in the sense that a particular window in time (in childhood or adulthood) had a specifically high impact on self-rated health since the effects from the two periods in time were very similar. Furthermore, stratifying the study population into six age groups showed similar odds of SRH with regard to exposure in adulthood in all age groups in both men and women.

Causal relationships in life course epidemiology are often complex. An important strategy to investigate causal mechanisms in process over long periods of time would be to investigate specific exposures and specific disease outcomes with reference to the accumulation, critical period and social mobility hypotheses. Childhood socioeconomic conditions seem to be connected with a major part of adult socioeconomic differences in mortality through consistent significant associations with cardiovascular diseases in adulthood [32,33]. It should also be noted that the three hypotheses are not necessarily mutually exclusive. Slow growth in childhood (critical period) for instance adds to occupational stress in a cumulative process (accumulation) to affect blood pressure in early old age [34].

Still, the patterns of general health status in a population in the form of self-rated health are also essential in relation to early life socioeconomic conditions [3,9]. Given the similar prevalence of poor self-rated health among men and women in this and other studies [35] and the considerably higher life expectancy among women than men in Sweden and other western countries, it may be that chronic diseases other than cardiovascular diseases with much lower mortality such as musculoskeletal and mental diseases are substantially affected by early life socioeconomic conditions such as economic stress in childhood. This study is the first to investigate the three hypotheses suggested by the life course approach to socioeconomic differences in health on self-rated health.

A life-course approach to health may provide a model to increase the understanding of how various exposures at different life-course stages can independently, cumulatively and through interaction influence health in adult life [36]. The collection of detailed data from various periods in life allows the identification of effects of exposure during specific time periods on a specific outcome. Previous studies which have investigated the three accumulation, critical period and social mobility hypotheses on cardiovascular [14,20] and all cause mortality [20] have used three observations over time during the life course including one in childhood when they tested the three hypotheses. Three observations is the strict requirement of a longitudinal study [37]. In our study, there is one observation in time (cross-sectional) with a recall item concerning economic stress in childhood, i.e. our study consists of references to only two observation points in time.

It should be noted that life course trajectories may not be linear. For example, by using Latent Class Growth Mixture Modelling three different life course trajectories have been observed for body mass index (BMI): a “normative” trajectory, a progressively overweight trajectory and a progressively overweight but stabilizing trajectory [38]. Still, this methodology is not applicable in our data material.

Socioeconomic differences have mostly been analysed in terms of socioeconomic status (SES) according to occupation, education or income [39]. Economic stress in childhood seems to be a not only plausible but also empirical factor to consider in the study of socioeconomic differences in childhood as a predictor of health in adulthood. Marital status of parent and growing up with two parents or a single parent [27] may be other important factors which have been previously less investigated, and which may be included in future surveys.

### Strengths and limitations

The response rate is approximately 55%. The group born outside Sweden is underrepresented in this investigation by approximately 4 per cent units compared to official register statistics for Skåne in southern Sweden. The distribution of demographic and social variables in a previous public health survey with a similar response rate conducted in Skåne in 2000 accorded well with the distribution of sociodemographic characteristics in the population of Skåne in 2000 in a comparison with official population registers [40], and comparisons for the 2008 investigation have yielded similar unpublished results. The risk of selection bias may thus be regarded as comparatively minor in our study.

Adult self report of economic stress in childhood most likely implies some element of recall bias. This recall bias would most probably lead to non-differential misclassification which would dilute and underestimate the results. Still, although our results are probably underestimated, they remain significant throughout the multiple regression analyses.

Confounders and potential mediating factors such as age, sex, country of origin, socioeconomic status, emotional support, instrumental support, trust, daily smoking and high alcohol consumption were controlled for by adjusting for these variables.

Self-rated health has previously been studied regarding the question of validity. This item is a good prospective predictor of for instance CVD incidence and mortality [25,26]. Previous Swedish studies have analyzed subjective economic hardships using the same item as in this study and demonstrated significant associations with health outcomes [41-43].

It is formally impossible to infer causality from cross-sectional studies. Also, in life course epidemiology three or more points in time are recommended if the aim is to investigate associations between risk factors in early life and health in adulthood, as in this study. Still, studies with cross-sectional study design may form at least some complementary part of conclusions concerning causal inference. One of the exposure variables in this study is a retrospective self reported item concerning economic stress in adolescence. Furthermore, it should be kept in mind that objective data concerning early life experiences of for instance economic stress in childhood (basic needs) are extremely scarce.

## Conclusions

The accumulation and social mobility hypotheses were confirmed. The critical period model was confirmed in the sense that both economic stress in childhood and economic stress in adulthood seemed to be of importance to self-rated health measured in adulthood. However, it was not confirmed in the sense that a particular window in time (in childhood or adulthood) had a specifically high impact on self-rated health.

## Competing interests

The authors declare that they have no competing interests.

## Author’s contributions

ML and MR have contributed to the conception and drafting of the work. ML has analysed the data and written the first draft of the manuscript. ML, KH and MR have contributed to the interpretation and the discussion of the results, and the revision of the content. All authors have read and approved the final manuscript.

## Pre-publication history

The pre-publication history for this paper can be accessed here:

http://www.biomedcentral.com/1471-2458/12/761/prepub

## References

[B1] Kuh D Ben-Schlomo Y A life course approach to chronic disease epidemiology 2004 2 Oxford: Oxford University Press

[B2] Bengtsson T Mineau GP Early-life effects of socioeconomic performance and mortality in later life: a full life-course approach using contemporary and historical sources Social Science and Medicine 2009 68 1561 1564 10.1016/j.socscimed.2009.02.012 19321248

[B3] Davey Smith G Lynch JW Kuh D, Ben-Schlomo Y Lifecourse approaches to ocioeconomic differentials in health A Life Course Approach to chronic disease epidemiology 2004 2 Oxford: Oxford University Press 77 115

[B4] Frankel S Davey Smith G Gunnell D Childhood socioeconomic position and adult cardiovascular mortality: the Boyd Orr cohort Am J Epidemiol 1999 150 1081 1084 10.1093/oxfordjournals.aje.a009932 10568623

[B5] Davey Smith G Hart C Upton M Hole D Gillis C Watt G Hawthorne V Height and risk of death among men and women: aetiological implications of associations with cardiorespiratory disease and cancer mortality J Epidemiol Community Health 2000 54 97 103 10.1136/jech.54.2.97 10715741 PMC1731616

[B6] Davey Smith G McCarron P Okasha M McEwen J Social circumstances in childhood and cardiovascular disease mortality: prospective observational study of Glasgow University students J Epidemiol Community Health 2001 55 340 340 10.1136/jech.55.5.340 11297656 PMC1731885

[B7] Davey Smith G Greenwood R Gunnell D Sweetnam P Yarnell J Elwood P Leg length, insulin resistance, and coronary heart disease risk: the Caerphilly study J Epidemiol Community Health 2001 55 867 872 10.1136/jech.55.12.867 11707479 PMC1731819

[B8] Lawlor DA Ebrahim S Davey Smith G Adverse socioeconomic position across the life course increases coronary heart disease risk cumulatively: findings from the British women’s heart and health study J Epidemiol Community Health 2005 59 9 785 793 10.1136/jech.2004.029991 16100318 PMC1733124

[B9] Hertzman C Power C Matthews S Manor O Using an interactive framework of society and life course to explain self-rated health in early adulthood Social Science and Medicine 2001 53 12 1575 1585 10.1016/S0277-9536(00)00437-8 11762884

[B10] Singh-Manoux A Martikainen P Ferrie J Zins M Marmot M Goldberg M What does self-rated health measure? Results from the British Whitehall II and French Gazal cohort studies J Epidemiol Community Health 2006 60 4 364 372 10.1136/jech.2005.039883 16537356 PMC2566175

[B11] Regidor E Pascual C Martinez D Ortega P Astasio P Calle ME Heterogeneity in the association between socioeconomic position in early life and adult self-rated health in two birth cohorts of Spanish adults J Epidemiol Community Health 2011 65 11 999 1005 10.1136/jech.2010.110601 21282139

[B12] Kermack WO McKendrick AG McKinley PL Death rates in Great Britain and Sweden: some regularities and their significance Lancet 1934 31 698 703

[B13] Barker DJP Mothers, babies and health in later life 1998 Edinburgh: Churchill Livingstone

[B14] Hallqvist J Lynch J Bartley M Lang T Blane D Can we disentangle life course processes of accumulation, critical period and social mobility? An Analysis of disadvantaged socio-economic positions and myocardial infarction in the Stockholm Heart Epidemiology Program Social Science and Medicine 2004 58 1555 1562 10.1016/S0277-9536(03)00344-7 14759698

[B15] Sahade V Franca S Badaro R Fernando Adan L Obesity and postprandial lipemia in adolescents: Risk factors for cardiovascular diseases Endocrinol Nutr 2012 59 2 131 139 10.1016/j.endonu.2011.08.004 22137533

[B16] Burdette AM Needham BL Neighborhood environment and body mass index trajectories from adolescence to adulthood J Adolesc Heal 2012 50 1 30 37 10.1016/j.jadohealth.2011.03.009 22188831

[B17] Mann S Wadsworth MEJ Colley J Accumulation of factors influencing respiratory illness in members of a national birth cohort and their offspring Journal of Epidemiology and Community Health 1992 46 286 291 10.1136/jech.46.3.286 1645088 PMC1059569

[B18] Wunsch G Duchene J Thiltges E Salhi M Socioeconomic differences in mortality. A life course approach Eur J Popul 1996 12 167 185 10.1007/BF01797082 12320553

[B19] Lynch JW Kaplan GA Cohen RD Kauhanen J Wilson TW Smith NL Salonen JT Childhood and adult socioeconomic status as predictors of mortality in Finland Lancet 1994 343 524 527 10.1016/S0140-6736(94)91468-0 7906766

[B20] Rosvall M Chaix B Lynch J Lindström M Merlo J Similar support for three different life course socioeconomic models on predicting premature cardiovascular mortality and all-cause mortality BMC Publ Health 2006 6 203 10.1186/1471-2458-6-203 PMC1569840 16889658

[B21] Lundberg O The impact of childhood living conditions on illness and mortality in adulthood Social Science and Medicine 1993 36 8 1047 1052 10.1016/0277-9536(93)90122-K 8475420

[B22] Elmén H Infant mortality: social inequality in a Swedish city Eur J Public Health 1993 3 237 241 10.1093/eurpub/3.4.237

[B23] Catalano R The health effects of economic insecurity Am J Public Health 1993 81 9 1148 1152 1951825 10.2105/ajph.81.9.1148 PMC1405640

[B24] Campbell A The sense of well-being in America: recent patterns and trends 1981 New York: McGraw-Hill

[B25] Heistaro S Jousilahti P Lahelma E Vartiainen E Puska P self-rated health and mortality: a long term prospective study in eastern Finland Journal of Epidemiology and Community Health 2001 55 227 232 10.1136/jech.55.4.227 11238576 PMC1731868

[B26] Moller L Kristensen TS Hollnagel H Self-rated health as a predictor of coronary heart disease in Copenhagen, Denmark Journal of Epidemiology and Community Health 1996 50 423 428 10.1136/jech.50.4.423 8882226 PMC1060313

[B27] Putnam RD Bowling Alone. The Collapse and Revival of American Community 2000 New York, London: Touchstone

[B28] Lindström M Marital status, social capital, material conditions and self-rated health: a population-based study Health Policy 2009 93 2–3 172 179 19692141 10.1016/j.healthpol.2009.05.010

[B29] Putnam RD Making Democracy Work. Civic Traditions in Modern Italy 1993 Princeton: Princeton University Press

[B30] Persson G Public Health Report 2005 2005 Stockholm: The National Board on Health and Welfare

[B31] Norusis MJ PASW for Windows. Advanced Statistics. Release 18.0 2011 Chicago: SPSS

[B32] Galobardes B Davey Smith G Jeffreys M McCarron P Childhood socioeconomic circumstances predict specific causes of death in adulthood: the Glasgow student cohort study J Epidemiol Community Health 2006 60 527 529 10.1136/jech.2005.044727 16698985 PMC2563940

[B33] Galobardes B Davey Smith G Lynch JW Systematic review of the influence of childhood socioeconomic circumstances on risk for cardiovascular disease in adulthood Ann Epidemiol 2006 16 91 104 10.1016/j.annepidem.2005.06.053 16257232

[B34] Montgomery S Berney L Blane D Prepubertal stature and blood pressure in early old age Arch Dis Child 2000 82 358 363 10.1136/adc.82.5.358 10799423 PMC1718317

[B35] Lindström M Social capital, the miniaturization of community and self reported global and psychological health Social Science and Medicine 2004 59 3 595 607 10.1016/j.socscimed.2003.11.006 15144768

[B36] Kuh D Ben-Shlomo Y Lynch J Hallqvist J Power C Life course epidemiology J Epidemiology Community Health 2003 57 778 783 10.1136/jech.57.10.778 PMC1732305 14573579

[B37] Singer JD Willett JB Applied Longitudinal Data Analysis. Modeling Change and Event Occurrence 2003 Oxford: Oxford University Press

[B38] Hoekstra T Barbosa-Leiker C Koppes LLJ Twisk JWR Developmental trajectories of body mass index throughout the life course: an application of Latent Class Growth (Mixture) Modelling Longitudinal and Life Course Studies 2011 2 3 319 330

[B39] Lynch J Kaplan J Berkman L, Kawachi I Socioeconomic position Social Epidemiolog 2000 Oxford, New York: Oxford University Press 13 35

[B40] Carlsson F Merlo J Lindström M Östergren PO Lithman T Representativity of a postal questionnaire survey in Sweden, with special reference to ethnic differences in participation Scand J Public Health 2006 34 2 132 139 10.1080/14034940510032284 16581705

[B41] Persson G Health in Sweden: The National Public Health Report 2001 2001 Oslo: Scandinavian University Press

[B42] Olivius G Östergren PO Hanson BS Lyttkens CH Parental economic stress. Evidence of an overlooked public health risk among Swedish families Eur J Public Health 2004 14 354 360 10.1093/eurpub/14.4.354 15542869

[B43] Fritzell S Burström B Economic strain and self-rated health among lone and couple mothers in Sweden during the 1990s compared to the 1980s Health Policy 2006 79 253 264 10.1016/j.healthpol.2006.01.004 16473438

